# Effects of Fe(OH)_3_ and MnO_2_ Flocs on Iron/Manganese Removal and Fouling in Aerated Submerged Membrane Systems

**DOI:** 10.3390/polym13193201

**Published:** 2021-09-22

**Authors:** Güler Türkoğlu Demirkol, Suna Özden Çelik, Sevgi Güneş Durak, Seren Acarer, Ender Çetin, Sultan Akarçay Demir, Neşe Tüfekci

**Affiliations:** 1 Department of Environmental Engineering, Faculty of Engineering, Istanbul University-Cerrahpasa, Avcilar Campus, Istanbul 34320, Turkey; ecetin@iuc.edu.tr (E.Ç.); sultan.akarcay@yandex.com (S.A.D.); nese@iuc.edu.tr (N.T.); 2 Department of Environmental Engineering, Corlu Engineering Faculty, Namik Kemal University, Corlu/Tekirdag 59860, Turkey; sunacelik@nku.edu.tr; 3 Department of Environmental Engineering, Faculty of Engineering-Architecture, Nevsehir Haci Bektas Veli University, Nevsehir 50300, Turkey; sgdurak@nevsehir.edu.tr

**Keywords:** Fe^2+^, Mn^2+^, removal, membrane, fouling

## Abstract

Many treatment methods are used to remove iron and manganese from water. Aeration and membrane filtration are two of these methods. In this study, Fe^2+^ and Mn^2+^ removal by aeration with different catalysts and instead of simple membrane filtration applied in other studies, the aerated-submerged membrane systems were evaluated separately. When Fe(OH)_3_ was applied in the aeration step and complete oxidation of Fe^2+^ was obtained after 27 min, while complete Mn^2+^ oxidation was obtained in 76 min. However, when MnO_2_ was applied in the aeration step, complete oxidation of Fe^2+^ and Mn^2+^ was relatively slow (36 and 110 min, respectively). According to the results obtained from the aerated membrane system, Fe^2+^ and Mn^2+^ removal were extended by Fe(OH)_3_ via adsorption/surface oxidation. It is clearly shown from the flux, resistance results, scanning electron microscope (SEM) and Fourier transform infrared (FT/IR) spectroscopy observation that manganese oxides were deposited mainly in membrane pores forming membrane fouling by small flocs, while iron oxide particles were deposited on the membrane surface. Although the flux performance of PT PES membrane was higher than HF PP membrane, fouling resistance of HF PP membrane was higher than PT PES.

## 1. Introduction

Iron and manganese removal from water sources is important for drinking and both domestic and industrial uses. The formation of MnO_2_, even at a concentration of 0.2 mg/L of manganese, causes the formation of black sludge in the inner walls of the pipe. According to the US Environmental Protection Agency (EPA) and European Union (EU) regulations, the allowed value for manganese is 0.05 mg/L [1]. The presence of dissolved, colloidal, and particulate iron and manganese in water varies greatly depending on the ambient pH and the amount of dissolved oxygen. The presence of organic matter and various anions in the environment are important factors that determine the type of iron and manganese oxide formed by aeration and its conversion over time [2,3,4]. Iron oxide minerals have a high specific surface area (>100 m^2^/g). Similarly, manganese oxide flocs have a large surface area. Therefore, they are effective adsorbents for many dissolved ions, molecules, and gases.

Various technologies are enriched and used in iron and manganese removal. Ion exchange, biological trickling filter, reverse osmosis, nanofiltration, and aeration are some of these methods [5]. In studies on the treatment of iron and manganese with aeration, it is stated that the reaction accelerated with the addition of Fe(OH)_3_; in other words, Fe(OH)_3_ flocs had a catalytic effect on Fe^2+^ oxidation [6]. O’Connor claimed that in the majority of iron and manganese removal facilities in the USA, aeration, retention tank/settling, and filtration are widely applied. They explained that Fe(OH)_3_ flock has a very high capacity to adsorb Fe^2+^, and this is explained by iron removal in contact filters as well as inside the filters, where the filter medium is covered with Fe(OH)_3_. They also stated that an aging process is required in the filter for the flocs that will replace the precipitate [7]. Takai pointed out that among many iron oxides, only γ-FeOOH is an effective catalyst [8]. Andersen et al. revealed that catalysts play an important role in the oxidation of iron and manganese [9]. They explained this by increasing the efficiency of multiple treatment plants after the formation of oxidized iron and manganese in an aeration or filter medium. The study of Coughlin and Matsui handled higher initial Mn^2+^ concentrations and revealed the catalytic effect of manganese oxides formed by the aeration on Mn^2+^ oxidation [10]. They also stated that manganese oxides catalyze the removal of Mn^2+^ by aeration and the increase in removal cannot be explained only by adhering to the manganese oxide surface. Sung investigated the effect of iron oxides on the removal of Mn^2+^ by aeration [11]. Accordingly, it is determined that iron oxide is a catalyst as effective as manganese oxide. Davies and Morgan stated that Mn^2+^ oxidation is faster in the presence of goethite (α-FeOOH) than in the presence of lepidocrocite (γ-FeOOH) or silicon oxide [12]. Tüfekci and Sarikaya observed that the catalytic effect of Fe^3+^ increased up to 600 mg/L and beyond this value, Fe^3+^ did not have a significant catalytic effect on the oxidation of Fe^2+^ [13]. In addition, it is observed that the catalytic effect increased up to three days with the aging of Fe(OH)_3_ sludge. It has been expressed that the increase of the catalytic effect with the aging of the sludge may cause the reaction of Fe^2+^ radicals to be accelerated in the reaction of Fe^2+^ with oxygen, and that any of the different structural forms of iron oxides can be effective in this reaction rate. Similar catalytic effects were observed up to 700 mg/L concentration for MnO_2_ for the oxidation rate of Mn^2+^ [14]. In the study by Ormancı et al., it is pointed out that MnO_2_ accelerated the Mn^2+^ oxidation up to 800 mg/L and that there is no significant effect beyond this value [15]. In the study of Gunes Durak et. al., it is stated that the catalytic effect increased up to four days with the aging of MnO_2_ sludge [16]. It is emphasized in the study conducted by Celik, although the removal of Mn^2+^ with aeration is quite slow at pH = 8.5, Mn^2+^ removal efficiency increased significantly if Fe(OH)_3_ and/or MnO_2_ is added [17]. Similar results were obtained by Ormanci and Turkoglu [18,19]. In the study conducted by Cheng, it is found that when dissolved oxygen is sufficient, iron and manganese are completely removed from the solution [20]. When dissolved oxygen is below 3 mg/L, only iron is removed, while manganese remained in solution. Various aeration systems are used in four different plans by Štembal et al. [21]. Dissolved oxygen ranges from 8–17 mg/L values. Groundwater iron concentrations used in the study were 0.98–2.45 mg/L. After treatment, the iron is reduced to a standard value of 0.3 mg/L in the filter at a depth of 0.8 m (Table 1).

The most important issue for iron and manganese removal in membrane systems is that the selected membrane is below the particle size of iron and manganese so that it can function to hold the particles. However, when compared with ceramic membranes, polymeric membranes provide up to 100% iron and manganese removal [5]. The particle size of Fe^2+^ and Mn^2+^ in dissolved form is too small to be kept by microfiltration and ultrafiltration. Ion exchanger, ultrafiltration (UF) membrane is tried for Fe^2+^ and Mn^2+^ removal, but it is determined that more than 74% of the parts passed through the membrane. Again, nanofiltration and reverse osmosis membrane are tried for Fe^2+^ and Mn^2+^ removal, and it is determined that reverse osmosis membrane did not meet the standards, although it provides better Mn^2+^ removal. Therefore, an oxidation process is essential before the membrane. While this oxidation process can be just simple aeration for Fe^2+^ at natural water pH, it requires a stronger oxidant for Mn^2+^ removal. Strong oxidants such as chlorine derivatives, potassium permanganate or ozone should be applied as oxidants. Iron and manganese oxides formed by oxidation can also contribute to the removal of turbidity and other pollutants, as they contain other pollutant particles in the water. In the work of Choo et al., while iron oxide particles do not cause fouling in the membrane, ultrafiltration is not sufficient for oxidized manganese particles and caused significant fouling in the ultrafiltration membrane [5]. The membrane used in the study is cellulose acetate and it has 100 kDa MWCO. In a study conducted by Kan et. al., microfiltration is applied following NaOCl oxidation for Fe^2+^ and Mn^2+^ removal [22]. Oxidized metal ion particles are examined with a particle counter. In the study, manganese values are reduced below the standards after two weeks of application. According to the results, it is concluded that the iron and manganese oxide layer deposited on the membrane surface had an important role in manganese removal. The membrane used in the study is made of PTFE material whose surface has been treated with a hydrophilic polymer. In their studies where Yu et al. compared the fouling properties of the PVDF membrane coated and uncoated with MnO_2_ nanoparticles, they determined that the membrane coated with MnO_2_ is less fouled, while the uncoated membrane is exposed to both recyclable and irreversible fouling [23]. According to the results of the work of Celik, iron oxides were found more effective than manganese oxides to remove Fe^2+^ and Mn^2+^ in both aeration and aerated-submerged membrane systems, and that significant iron and manganese removal efficiencies were obtained if both oxides were present in the solution [17]. Iron oxides also provided significant iron, manganese, and TOC removal efficiencies. Based on this, she stated that iron oxides increase the lifetime of the membrane and that it can be recycled by recycling or chemical cleaning rather than irreversible fouling. As a result of the study, it has been determined that Fe(OH)_3_ increases Fe^2+^ and Mn^2+^ removal efficiency through adsorption/oxidation on the surface, and also that the flocs it produces grows beyond the membrane and cause an increase in membrane productivity. Similarly, it is stated that Fe(OH)_3_ caused a decrease in pressure increase, which is an indicator of membrane fouling and an increase in removal efficiency [18] (Table 1).

When Table 1 is examined, there is an increase in the removal efficiency when catalysts such as MnO_2_, FeO, α-FeOOH, and Fe(OH)_3_ are used for the aeration method in iron and manganese removal. Membrane filtration method enriched with oxidants also provides high removal efficiency in treatment.

In this study, Fe^2+^ and Mn^2+^ removal by aeration and the aerated-submerged membrane systems were investigated experimentally. MnO_2_ and Fe(OH)_3_ were used as catalysts in order to remove iron and manganese in the aeration method. The pH was adjusted as 6.5 for iron removal and 9.2 for manganese removal. According to these values, the effects of different doses of oxidants on the oxidation time were determined. In the ventilated submerged membrane method, Fe^2+^, Mn^2+^, and Fe^2+^-Mn^2+^ removal were investigated with plate-type polyethersulfone (PES) and hollow fiber polypropylene (PP) membranes. The flux performance and fouling resistance of the membranes were determined. Clean membranes and after the Fe^2+^, Mn^2+,^ and Fe^2+^-Mn^2+^ experiments the contaminated membranes were characterized by FT/IR and SEM, and the effect of iron and manganese on membrane fouling was determined.

**Table 1:** Studies in the literature on iron and manganese removal by aeration and/or filtration.

Removal Process	Ambient	Results	Reference
Aeration + Filtration	FeCOH_3_ covered filter	High-capacity Fe^2+^ adsorption	O’Connor [7]
Aeration + Filtration	Catalysts	Effective iron and manganese removal	Andersen [9]
Aeration	Catalysts—MnO_2_	Effective manganese removal	Coughlin &Matsu [10], Sung [11], Tüfekci and Sarikaya [13], Güneş-Durak et al. [16]
Aeration	Catalysts—FeO	Effective iron removal	Sung [11]
Aeration	Catalysts—α-FeOOH	Fast oxidation	Davies and Morgan [12]
Aeration	Catalysts—Fe(OH)_3_/MnO_2_	Effective manganese removal	Çelik [17], Türkoglu [18,19]
Aeration	Sufficient dissolved O_2_	Completely manganese and iron removal	Cheng [20]
Biological trickling filter	(Absence iron and ammonia)	94% manganese removal	Gouzinis et al. [24]
Reverse Osmosis-UF (Dead end)	Oxidant- Chlorine	80% manganese removal	Choo et al. [5]
MF	Oxidant- NaOCl	90% manganese removal	Kan et al. [22]
Aeration + MF	-	99% iron and manganese removal	Celik [17]
Aeration + UF	-	99% iron and manganese removal	Celik [17]

## 2. Materials and Methods

### 2.1. Experimental Setup of Oxidation

**Figure 1:**
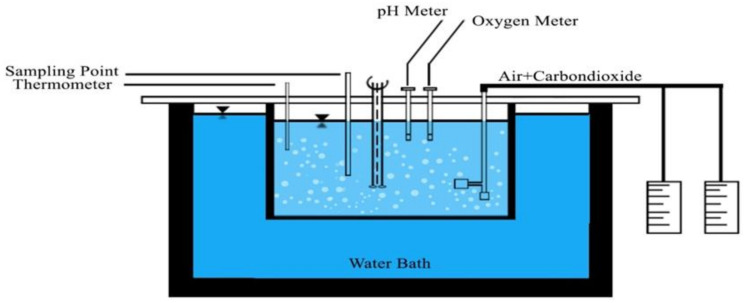
Experimental set up for oxidation with aeration.

The effect of iron and manganese oxides on the oxidation of Fe^2+^ and Mn^2+^ by aeration is studied in a laboratory scale batch reactor (Tin Mühendislik, Istanbul, Turkey) of 2 L volume under the constant pH, temperature, alkalinity, and O_2_ concentration. The experimental setup is illustrated in Figure 1. The solution is continuously mixed with the WiseStir HS-50A model of a mechanical mixer (Witeg, Wertheim, Germany). NaHCO_3_ is added into the solution to obtain 2 × 10^−2^ eq/L alkalinity. Air and CO_2_ are given into the solution using fine bubble diffusers (KHN, Yixing, China). The pH of the solution is controlled by adjusting the flow of CO_2_ gas. Since the HCO_3_-CO_2_ buffer system is used, the pH is adjusted by changing the CO_2_. HACH HQ40d type pH meter (HACH Company, Loveland, CO, USA) is used for temperature and pH measurement. The dissolved oxygen levels are monitored using the Armfield oxygen meter (Armfield Limited, Ringwood, UK). Constant temperature (25 °C) is maintained by immersing the reaction tank into GN 111–200 Gastronorm water bath (Gastronorm, Ponte nelle Alpi, Italy). Fe^2+^ stock solution is prepared by dissolving Ferro ammonium sulfate in 1 L demineralized water containing 2 mL of concentrated H_2_SO_4_. The samples taken at predecided times as measured from the start of the experiments are transferred into the 25 mL flasks containing 1 mL of (1 + 4) H_2_SO_4_. The determination of Fe^2+^ is carried out by spectrophotometric determination of Fe^2+^ with 1.10 phenanthroline in the presence of a large amount of Fe^3+^ given by Tamura and Goto [25] with PC Instrument T80 UV/VIS (PG Instruments Limited, Leicestershire, UK) model spectrophotometer.

Fe^2+^ and Mn^2+^ removal in the aeration step was evaluated at pH = 6.5 and 9.2, respectively. The pH of the solution is controlled by adding 0.1N NaOH/H_2_SO_4_ for Mn^2+^ oxidation. Mn(II) stock solution is prepared by dissolving manganese sulfate monohydrate (MnSO_4_·H_2_O) in 1 L demineralized water. The samples taken at predetermined times are immediately filtered and acidified after filtration with 2 mL HNO_3_. The detection limit for the AAS manganese measurement is 0.015 mg/L as Mn(II). All experiments are conducted at 25 °C and 9.2 of pH, 2 × 10^−2^ eq/L of alkalinity. Mn^2+^ measurement is performed according to the Standard Methods (3010A). The sample is filtered through a 0.22 µm filter before analysis.

### 2.2. Experimental Setup of Submerged Membrane System

**Figure 2:**
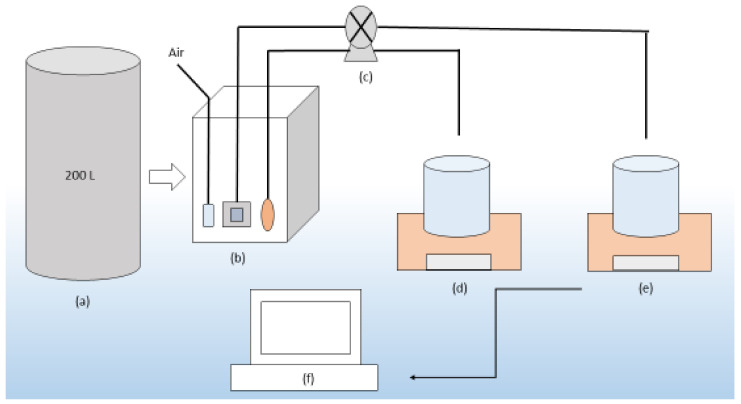
Experimental setup for submerged membrane system: (**a**) Feeding tank; (**b**) Reactor and submerged membranes; (**c**) Peristaltic pump; (**d**) Scales (PES membrane filtration weighing); (**e**) Scales (PP membrane filtration weighing); and (**f**) Computer.

**Figure 3:**
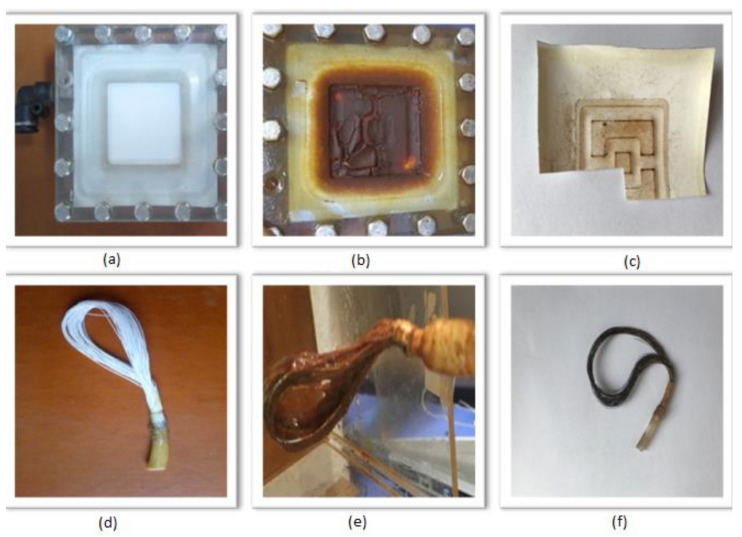
Clean and fouled membranes images. (**a**) Clean PES membrane; (**b**) fouled PES membrane after Fe^2+^ removal experiments; (**c**) fouled PES membrane after Mn^2+^ removal experiments; (**d**) clean PP membrane; (**e**) fouled PP membrane after Fe^2+^ removal experiments; (**f**) fouled PP membrane after Mn^2+^ removal experiments.

Polyethersulfone and polypropylene materials are among the most used polymeric membrane materials [26,27,28,29,30]. In addition, the hollow fiber membrane has a higher packing density. For these reasons, plate type polyethersulfone and hollow fiber polypropylene membrane were found suitable for comparison. Plate type polyethersulfone (PT PES) and hollow fiber polypropylene (HF PP) membrane are used in the submerged membrane system setup. The experimental setup consists of a 200 L volume polyethylene feed tank, 10 cm × 10 cm × 45 cm plexiglass reactor feeding by a peristaltic pump. Experiments are conducted with synthetic solutions. During the experiment, the air is fed from the bottom of the reactor by using fine bubble diffusers. Flux is continuously measured with scales and is controlled at constant pressure. The schematic diagram of the submerged membrane experimental setup is presented in Figure 2. Determination of Fe^2+^ and Mn^2+^ is done with the same methods as in oxidation analysis [25,31]. The physical and chemical properties of the membranes are given in Table 2 and images are presented in Figure 3.

Removal of Fe^2+^, Mn^2+^, and Fe^2+^-Mn^2+^ in submerged membrane reactor (Tin Mühendislik, Istanbul, Turkey) is studied for 90 days for each case. The pH is adjusted to 6.5, 9.5, and 8.5, respectively. Hollow fiber PP membrane is used by combining approximately 15 fibers to make the plate type PES equal to the surface area of the membrane. After the analyzes, samples are taken from fouled membranes. Images of membranes before and after experiments are given in Figure 3. While fouled membranes with Fe^2+^ experiments show dark reddish color on the membrane surface, Mn^2+^ flocks give the membrane a dark blackish appearance.

## 3. Results and Discussion

### 3.1. Oxidation Results

#### 3.1.1. Catalytic Effect of MnO_2_ and Fe(OH)_3_ on Oxidation of Fe^2+^ by Aeration

**Figure 4:**
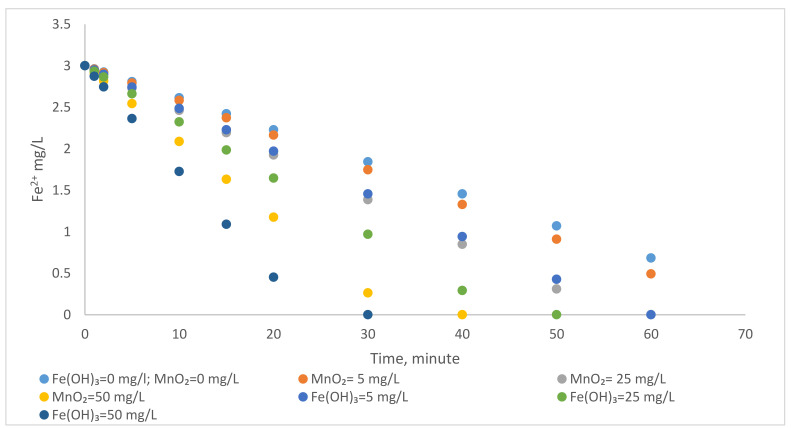
The effect of MnO_2_ and Fe(OH)_3_ on the oxidation of Fe^2+^ with atmospheric oxygen (Fe^2+^ = 3 mg/L, pH = 6.5, alkalinity = 2 × 10^−2^ eq/L, temperature = 25 °C).

The change in the rate of Fe^2+^ oxidation with atmospheric oxygen is investigated by adding Fe(OH)_3_ and MnO_2_ to the medium separately. Figure 4 shows the catalytic effects of MnO_2_ and Fe(OH)_3_ on the oxidation of Fe^2+^. The reaction is completed in 79 min without adding MnO_2_ and Fe(OH)_3_ (Table 3). It is observed that the reaction completion time is reduced to 36 min and 27 min when 50 mg/L MnO_2_ and 50 mg/L Fe(OH)_3_ are added to the reactor, respectively. The homogeneous rate constant, k, is determined as 0.038 min^−1^. In the case of adding MnO_2_ and Fe(OH)_3_ to the reactor, it is observed that the value of the heterogeneous rate constant (kcat) is 0.08 min^−1^ and 0.107 min^−1^, respectively.

It is seen from Figure 4 that MnO_2_ and Fe(OH)_3_ flocs added to the medium accelerate oxidation. It is also observed that the catalytic effect of Fe(OH)_3_ on the oxidation of Fe^2+^ is higher than MnO_2_ (Table 3 and Figure 4). It is thought that the reason for this is due to the characteristics of Fe(OH)_3_ and MnO_2_. Fe(OH)_3_ may be more effective in the floc formation in the reactor. Kasim et al. found that MnO_2_ is more stable than Fe(OH)_3_ when the pH > 8 [32]. Therefore, the higher reaction time required for removal when using MnO_2_ may depend on the stability of MnO_2_.

#### 3.1.2. Catalytic Effect of MnO_2_ and Fe(OH)_3_ on Oxidation of Mn^2+^ by Aeration

**Figure 5:**
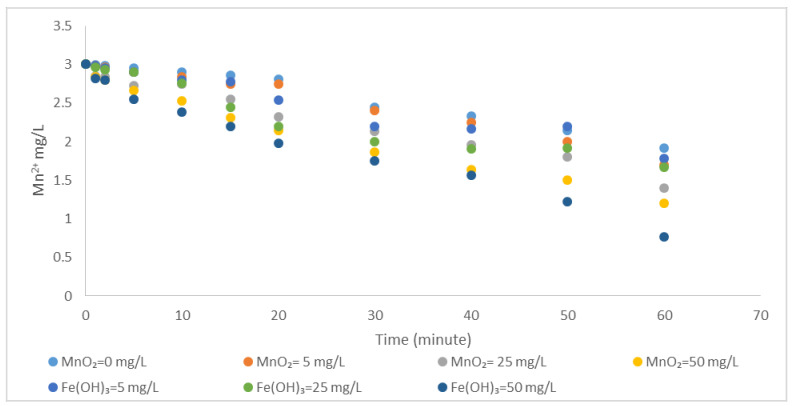
The effect of MnO_2_ and Fe(OH)_3_ on the oxidation of Mn^2+^ with atmospheric oxygen (Mn^2+^ = 3 mg/L, pH = 9.2 alkalinity = 2 × 10^−2^ eq/L, temperature = 25 °C).

The variation in the oxidation rate of Mn^2+^ is investigated by adding MnO_2_ and Fe(OH)_3_ to the medium separately. The catalytic effect of MnO_2_ and Fe(OH)_3_ on the oxidation of Mn^2+^ is shown in Figure 5 and Table 4. When Table 4 is examined, it is seen that the reaction completion time is 177 min without adding MnO_2_ and Fe(OH)_3_ to the medium It was observed that the reaction completion time was reduced to 110 min when 50 mg/L MnO_2_ was added to the reactor, and the completion time of the reaction was reduced to 76 min when 50 mg/L Fe (OH)_3_ was added. The homogeneous rate constant, k is determined as 0.0169 min^−1^. When MnO_2_ is added to the medium, the rate constant (kcat) is obtained as 0.0272 min^−1^. The rate constant (kcat) is obtained as 0.0392 min^−1^ if Fe(OH)_3_ is added to the medium.

As can be seen from the results, MnO_2_ and Fe(OH)_3_ flocs addition to the medium accelerated the oxidation of Fe^2+^and Mn^2+^. It is also observed that the catalytic effect of Fe(OH)_3_ is higher than MnO_2_. In general, it is determined that the catalytic effect of Fe(OH)_3_ on the oxidation of Fe^2+^ and Mn^2+^ with atmospheric oxygen is higher than that of MnO_2_. This phenomenon can be attributed to the positive charge of Fe(OH)_3_ flocs within the pH ranges of the study since the point of zero charges of iron oxide is in the range of 8.5–9.3 [17]. As a result, the diffuse layer of Fe(OH)_3_ flocs will have negatively charged hydroxide ions rather than hydrogen ion and as a result, the pH value in the particle layer are higher than the solution pH. Since Fe^2+^ oxidation rate is known to be the second-order of OH-dependence, Fe(OH)_3_ flocs accelerate oxidation due to high pH in their scattered layers [33,34,35].

### 3.2. Submerged Membrane Filtration Results

Submerged membrane experiments were conducted at pH = 8.5, representing the average pH of aeration tank of water treatment plants as well as the same pH of oxidation experiments (6.5 for Fe^2+^ and 9.2 for Mn^2+^) to present membrane fouling.

#### 3.2.1. Removal of Fe^2+^ and Mn^2+^

**Figure 6:**
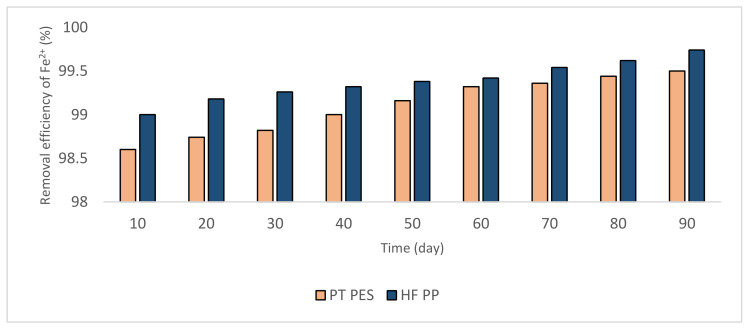
Removal efficiencies obtained from Fe^2+^ experiments (Fe ^2+^ = 3 mg/L, pH = 6.5, alkalinity = 2 × 10^−2^ eq/L).

**Figure 7:**
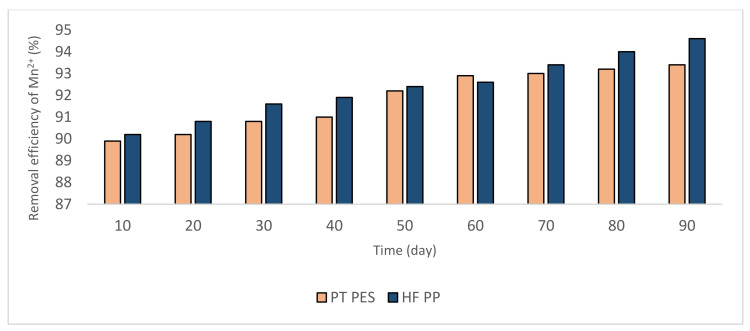
Removal efficiencies obtained from Mn^2+^ experiments (Mn^2+^ = 3 mg/L, pH = 9.2, alkalinity = 2 × 10^−2^ eq/L).

In the study, two types of membranes, plate type polyethersulfone (PT PES) and hollow fiber polypropylene (HF PP) membrane are used in the submerged membrane system setup. During the experiment, permeate samples are taken every 10 days during 90 days of operating period and the analysis of Fe^2+^ and Mn^2+^ is conducted according to the methods explained in the Materials and Methods Section. Based on the obtained results were given in Figure 6 and Figure 7, although high removal efficiencies were obtained (≥90%), Fe^2+^ removal efficiency was higher than Mn^2+^. During the operation, it was seen that the removal efficiency increased during the operating period. The major part of Fe^2+^ and Mn^2+^ is oxidized by feeding air. It is thought that the remaining unoxidized part of Fe^2+^ and Mn^2+^ are removed by adsorption onto iron oxide and manganese hydroxide flocs on the membrane surface. However, it is determined that iron oxide causes minimal membrane fouling and the water quality has not possessed a notable impact on the to extend of fouling [36]. These phenomena can explain obtained high iron removal efficiencies.

**Figure 8:**
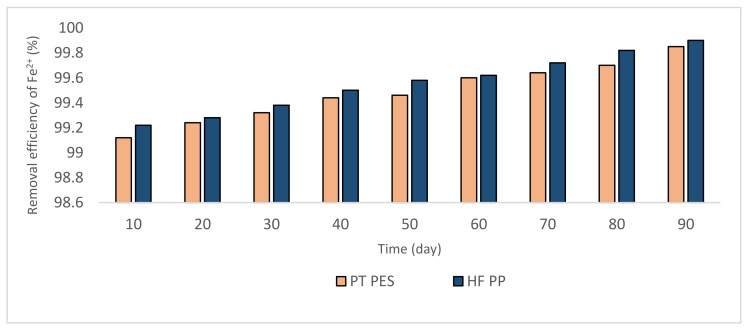
Fe^2+^ removal efficiencies obtained from Fe^2+^-Mn^2+^ experiments (Fe^2+^ = 3 mg/L, pH = 8.5, alkalinity = 2 × 10^−2^ eq/L).

**Figure 9:**
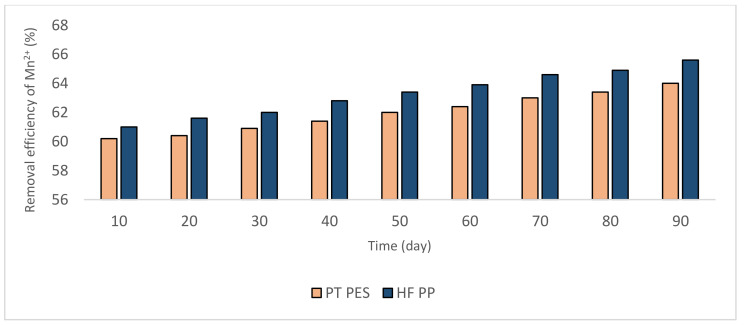
Mn^2+^ removal efficiencies obtained from Fe^2+^-Mn^2+^ experiments (Mn^2+^ = 3 mg/L, pH = 8.5, alkalinity = 2 × 10^−2^ eq/L).

Figure 8 and Figure 9 give the Fe^2+^-Mn^2+^ removal results. These experiments were conducted at pH = 8.5. The removal efficiency increased with the catalytic effect of the oxides, the amount of which increased with time (Figure 8 and Figure 9). According to the results, obtained Mn^2+^ removal efficiency is notably lower (60–65%) than that of pH = 9.2 experiments (90–95%). Nevertheless, Fe^2+^ removal efficiency is ≥99%.

According to the literature, oxidation kinetics of Mn^2+^ by aeration at pH = 8.5 is very slow (20–30%) [17,18,37]. In these experiments, this rate is raised up to 60–65% with the contribution of Fe^2+^.

#### 3.2.2. Membrane Flux

**Figure 10:**
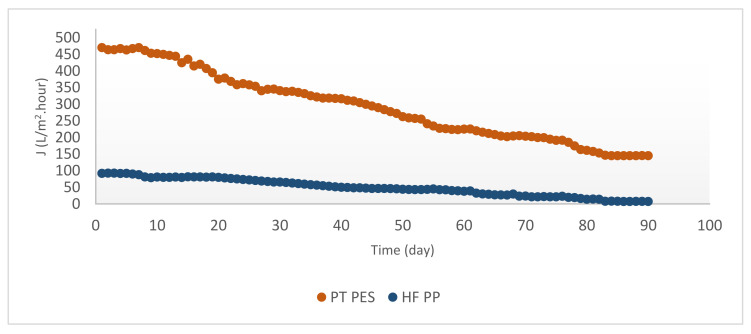
Flux versus time for Fe^2+^ removal experiments (Fe^2+^ = 3 mg/L, pH = 6.5, alkalinity = 2 × 10^−2^ eq/L).

**Figure 11:**
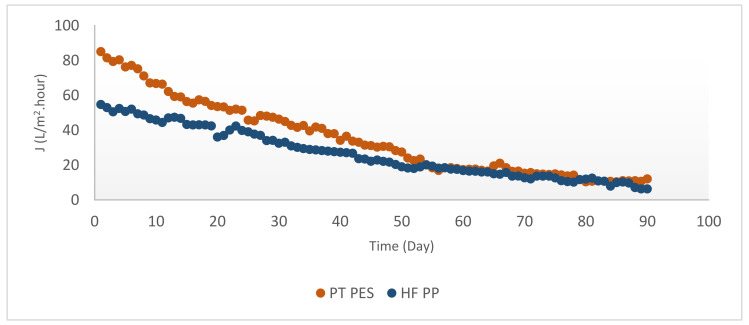
Flux versus time for Mn^2+^ removal experiments (Mn^2+^ = 3 mg/L, pH = 9.2, alkalinity = 2 × 10^−2^ eq/L).

**Figure 12:**
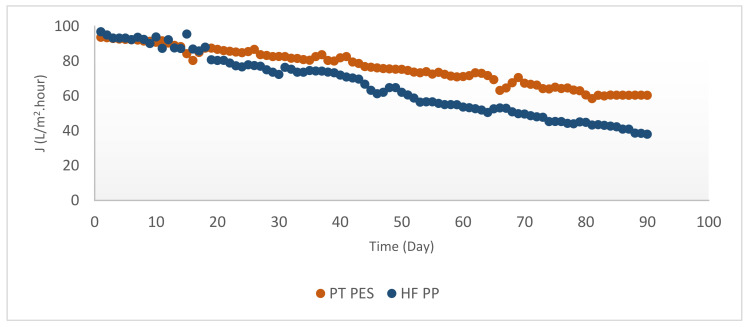
Flux versus time for Fe^2+^-Mn^2+^ removal experiments (Fe^2+^ = 3 mg/L, Mn^2+^ = 3 mg/L, pH = 8.5, alkalinity = 2 × 10^−2^ eq/L).

Removal of Fe^2+^, Mn^2+^, and Fe^2+^-Mn^2+^ in the PT PES and HF PP submerged membrane reactor is studied for 90 days for each case. The effect of iron and manganese oxides on membrane flux was investigated. Steady-state fluxes were calculated from the average flux of the last 10 days Flux variation obtained from experiments with the submerged membrane during 90 days is given in Figure 10, Figure 11, Figure 12. According to Figure 10, initial flux was J_0_ = 470.00 L/m^2^·h, while steady-state flux J_d_ = 148.57 L/m^2^·h with PT PES membrane. Obtained initial and steady-state fluxes of HF PP membrane were J_0_ = 91.79 L/m^2^·h and J_d_ = 9.48 L/m^2^·h, respectively. It is found that PT PES membrane have higher initial and steady-state flux than that of HF PP membrane. As shown from Figure 10, flux initially declined with time for both types of membranes.

According to Figure 11, flux initially declined with time for both types of membranes. Even though PT PES has a higher initial flux than HF PP membrane, during the Mn^2+^ removal experiments, both membranes showed similar steady-state flux characteristics. Nevertheless, PT PES membrane gave a higher permeate flux compared with HF PP membrane. Obtained initial and steady-state fluxes of HF PP membrane were J_0_ = 54.70 L/m^2^·h and J_d_ = 9.37 L/m^2^·h, respectively. Initial and steady-state flux of PT PES membrane were found as J_0_ = 84.96 L/m^2^·h and J_d_ = 10.81 L/m^2^·h, respectively [38].

As it shown from Figure 12, both membranes used in this study had similar initial flux trend for Fe^2+^-Mn^2+^ removal experiments, even though differentiated with time.

Obtained initial and steady state fluxes of PT PES membrane were J_0_ = 93.69 L/m^2^·h and J_d_ = 60.22 L/m^2^·h. Initial and steady state fluxes of HF PP membrane were recorded as J_0_ = 96.85 L/m^2^·h and J_d_ = 41.53 L/m^2^·h. Obtained initial and steady-state fluxes were shown in Table 5. According to the table, in every case obtained fluxes with PT PES membrane were relatively higher than HF PP membrane. It can be explained by the PT PES permeability pattern due to its membrane structure. Relatively high permeate flux obtained in Fe^2+^ experiments can be explained by hindering pore-blocking through the accumulation of iron hydroxide on the PT PES membrane surface [17]. Initial permeate flux values obtained by PT PES membrane can be ordered as follows: J_0_ (Fe^2+^) > J_0_ (Fe^2+^-Mn^2+^) > J_0_ (Mn^2+^). Recorded highest permeate flux was obtained in Fe^2+^ experiments, while lowest permeate flux was obtained in Mn^2+^ experiments. This phenomenon can be explained by the pore-blocking effect of manganese hydroxide [5] and the accumulation of iron hydroxide on the membrane surface hindering pore-blocking as stated above. As clearly shown from Table 5, initial permeate flux values obtained by HF PP membrane can be ordered as follows: J_0_ (Fe^2+^-Mn^2+^) > J_0_ (Fe^2+^) > J_0_ (Mn^2+^) Recorded highest permeate flux (96.85 L/m^2^·h) was obtained from Fe^2+^-Mn^2+^ experiments by HF PP membrane. This phenomenon can be explained by the positive effect of iron hydroxide on membrane flux [17].

Overall, a better performance was obtained from the PT PES membrane. PES polymer is hydrophilic while PP polymer is hydrophobic polymer. Hydrophilic membranes have a high filtration rate and are less prone to fouling. Therefore, less adhesion occurred in the hydrophilic membrane pores and higher filtration and purification efficiency was obtained [39,40,41].

#### 3.2.3. Membrane Resistance

Resistance values were calculated using Equations (1)–(5). Resistance values of submerged PT PES and HF PP membrane obtained from Fe^2+^, Mn^2+^, and Fe^2+^-Mn^2+^ removal experiments were given in Table 6. Total resistance (*R_t_*), membrane resistance (*R_m_*), pore resistance (*R_p_*), and cake resistance (*R_c_*) values were calculated for each membrane performed in the experiments [42].

(1) $$R_{t}=\Delta P/\left(\;J\times \mu \right)$$

(2) $$R_{m}+R_{p}\;=\;\Delta P/\left(J_{waste\;water}+J_{pure\;water}\right)\times \mu$$

(3) $$R_{m}\;=\;\Delta P/\left(J_{pıure\;water}\times \mu \right)$$

(4) $$R_{p}\;=\left(R_{m}+R_{p}\right)-R_{m}$$

(5) $$R_{c}\;=R_{t}\;-\left(R_{m}+R_{p}\right)$$

The obtained results showed that overall resistance varied from 1.94 × 10^12^ to 30.73 × 10^12^ m^−1^, pore resistance from 0.26 × 10^12^ to 9.57 × 10^12^ m^−1^ and cake resistance from 0.96 × 10^12^ to 19.83 × 10^12^ m^−1^. Membrane resistance of PT PES and HF PP is 0.72 × 10^12^ m^−1^ and 1.33 × 10^12^ m^−1^, respectively. The membrane resistance is affected by several factors such as membrane porosity, membrane material, membrane pore size, and membrane thickness (besides the composition of solution) [29]. The contribution of membrane resistance to the overall resistance varied in the range of 2.70–37.11%, pore resistance from 13.40–31.14%, cake resistance from 49.49–69.26%. According to Maximus et al., cake resistance is more representative than the modified serial resistance model to reflect the actual fouling mechanism [39].

When the overall resistances were compared, it is shown that the overall resistance of HF PP membrane was higher than the PT PES membrane for Fe^2+^ removal experiments. The same trend had been observed for membrane resistance. In view of cake resistance, it is found that the HF PP membrane had higher cake resistance than the PT PES membrane. High cake resistance induces low initial flux [43]. It is clearly shown from Figure 10 and Figure 11, an initial flux of the HF PP membrane was found lower than that of the PT PP membrane.

All resistance parameters (overall, membrane, pore, and cake) of the HF PP membrane were found higher than the PT PES membrane for Mn^2+^ removal experiments and Fe^2+^-Mn^2+^ removal experiments.

**Figure 13:**
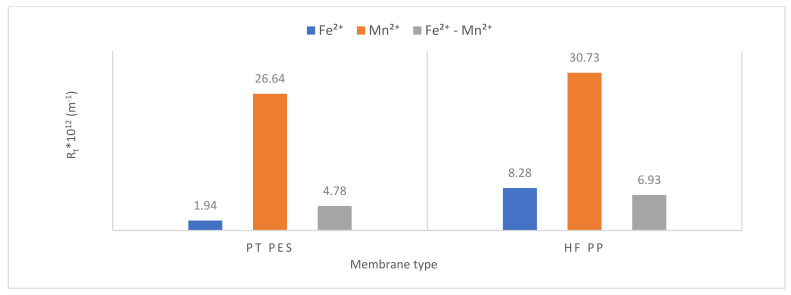
Overall resistance variation.

It is shown from Figure 13 that high overall resistance values were obtained with both membrane types for Mn^2+^ experiments. When the membranes were compared, it has been clearly shown that the highest overall resistance values were obtained with the HF PP membrane. This is related to the higher flux that can be achieved with the PT PES membrane than the HF PP membrane.

**Figure 14:**
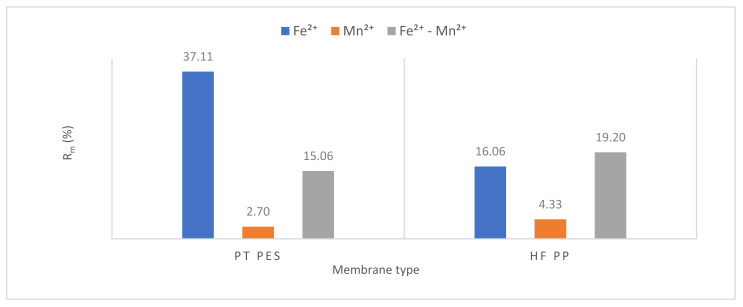
Membrane resistance variation.

As can be seen from Figure 14, the highest membrane resistance was obtained with PT PES membrane for Fe^2+^ removal experiments. Low membrane resistance values were obtained with both membranes for Mn^2+^ removal experiments. The presence of iron caused an increase in membrane resistance values. This suggests that iron oxide formed to have a positive effect on membrane resistance. HF PP membrane is less hydrophilic than PT PES membrane, for this reason, as can be seen from Figure 14, its membrane resistance is relatively lower. For HF PP membrane, the highest membrane resistance value has been obtained in Fe^2+^-Mn^2+^ removal experiments and the lowest value in Fe^2+^ removal experiments.

**Figure 15:**
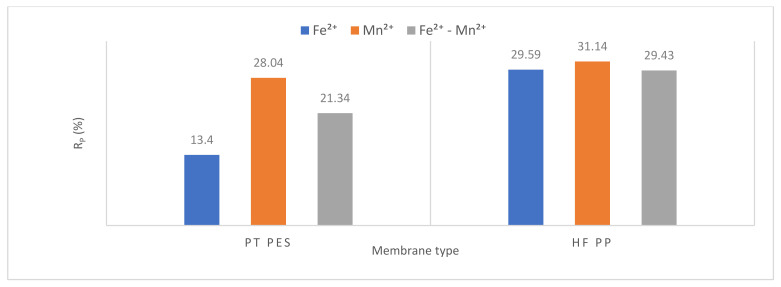
Pore resistance variation.

According to Figure 15, the highest pore resistance value was obtained in Mn^2+^ removal experiments with both membranes. The lowest pore resistance value was obtained in Fe^2+^ removal experiments for the PT PES membrane. On the other hand, the lowest pore resistance value for HF PP membrane was obtained in Fe^2+^-Mn^2+^ experiments. This can be explained by the fact that formed iron oxides accumulate on membrane surfaces rather than pores. An increase in pore resistance was observed in Mn^2+^ removal experiments. Unlike iron oxide, manganese oxide accumulates in membrane pores, which reduces the flux and increases the pore resistance. The overall resistance increases in Mn^2+^ removal experiments and less removal efficiency can be obtained compared to Fe^2+^ [21].

**Figure 16:**
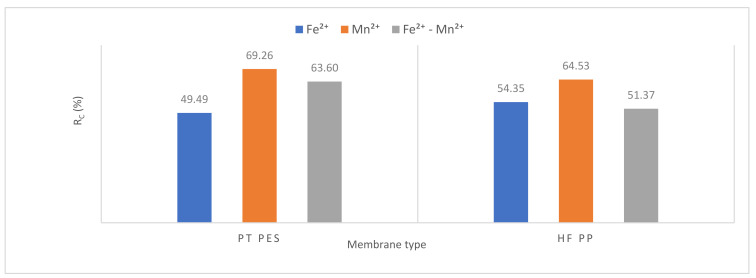
Cake resistance variation.

Cake resistance is one of the parameters that play an active role in membrane fouling and lifetime. As can be seen from Figure 16, cake resistance reached the highest value for Mn^2+^ removal experiments. The lowest flux has been also obtained for Mn^2+^ removal experiments. High cake resistance and low flux correspond to each other. The lowest cake resistance has been obtained in the Fe^2+^ removal experiment by PT PES membrane, and in the Fe^2+^-Mn^2+^ removal experiment by HF PP membrane. These results reveal that cake resistance plays an important role in membrane fouling and flux [44].

### 3.3. FT/IR Analysis

**Figure 17:**
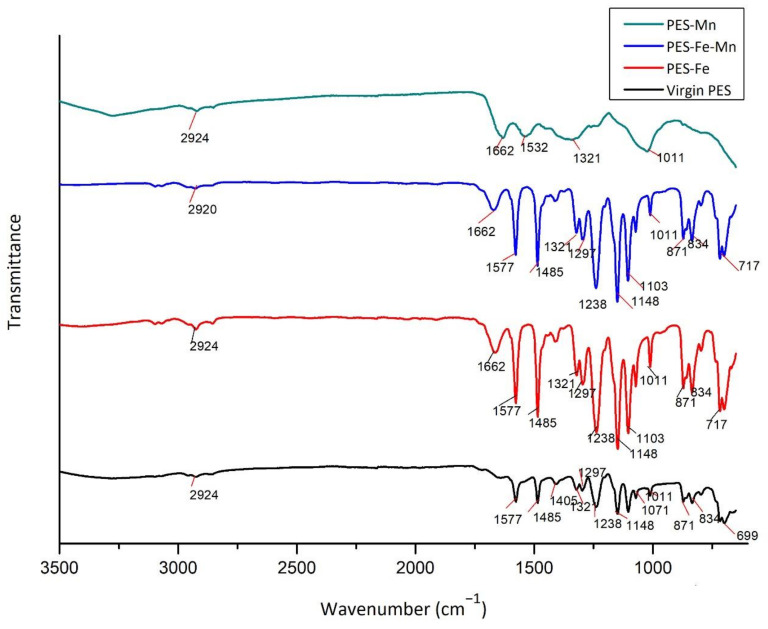
FT/IR spectra of fouled and virgin PT PES membrane.

**Figure 18:**
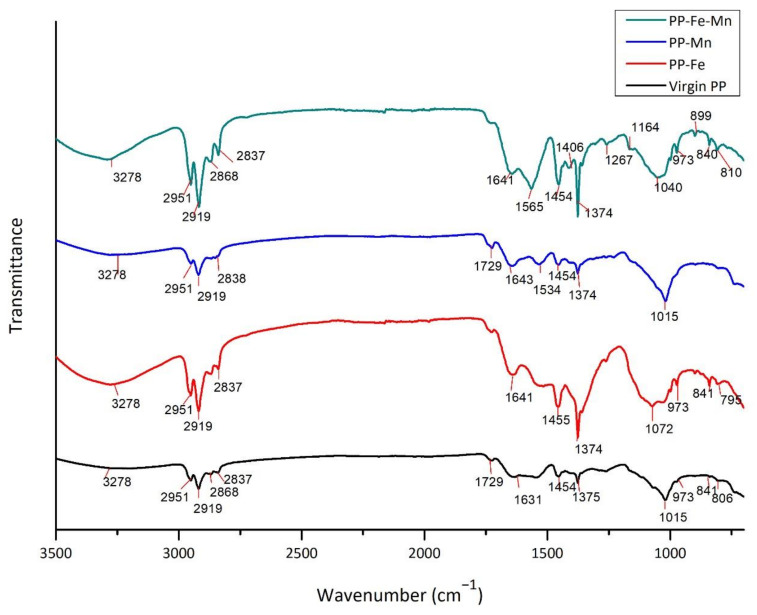
FT/IR spectra of fouled and virgin HF PP membrane.

Attenuated Total Reflection (ATR)-Fourier Transform Infrared (FTIR) Spectroscopy Spektrum-100 FT/IR (Perkin Elmer, Waltham, MA, USA) analyses were performed on the fouled and virgin membrane. ATR/FTIR spectra of fouled and virgin PT PES and HF PP membranes are shown in Figure 17 and Figure 18.

When the infrared spectrum of fouled and virgin PT PES membranes is examined, peaks observed at 2924 cm^−1^ wavenumber are assigned to O-H stretching. This group is attributed to the occurrence of membrane-air interaction [45]. The characteristic peaks originated from the vibration of aromatic bonds of PT PES membrane were observed from the appearance of the band at 1662 cm^−1^ and 1405 cm^−1^ range [46]. In the range of 1321 cm^−1^ to 1238 cm^−1^ peaks originated from C-O-C stretching. Peaks at 1148 cm^−1^ to 1011 cm^−1^ range were attributed to stretching of O=S=O bonds [47].

When the infrared spectrum of fouled PT PES membrane is examined, certain peaks shown in the virgin membrane disappeared and are replaced by new peaks. The peak observed at 1662 cm^−1^ for all fouled membranes is thought due to iron and manganese oxide presence [48]. When FTIR results were compared, a noticeable reduction in PT PES membrane peaks for Mn^2+^ removal experiments is observed. This can be explained by the fact that manganese oxide increases fouling due to smaller floc formation than iron oxide.

The virgin and fouled HF PP membrane spectrum shows the occurrence of a new peak at 2951 cm^−1^. This peak is indicative of CH_2_ vibrational bands [49,50] of HF PP polymer [51]. The band at 3278 cm^−1^ in the spectrum of HF PP membrane indicates O-H stretching possibly caused by adsorbed H_2_ O [52]. A broadband emerged at 1454 and 1374 cm^−1^ [53]. The peak at 1454 cm^−1^ originated from CH_3_ asymmetric deformation or CH_2_ bending vibration. The adsorption at 1374 cm^−1^ was due to CH_3_ symmetric deformation vibration [54]. The peak at 1015 cm^−1^ was assigned to the C-C asymmetric stretching, CH_3_ asymmetric wagging, and C-H wagging vibration [55].

When the infrared spectrum of fouled HF PP membrane used in Mn^2+^ experiments is examined, it can be seen that certain peaks disappeared shown in membranes of Fe^2+^ experiments and virgin membrane. This was due to the strong adsorption of manganese dioxide to the membrane inner layer. It is thought that iron oxide flocs are more adsorbed on membrane surfaces and acts as secondary membrane layers by adsorbing other pollutants.

### 3.4. SEM Analysis

**Figure 19:**
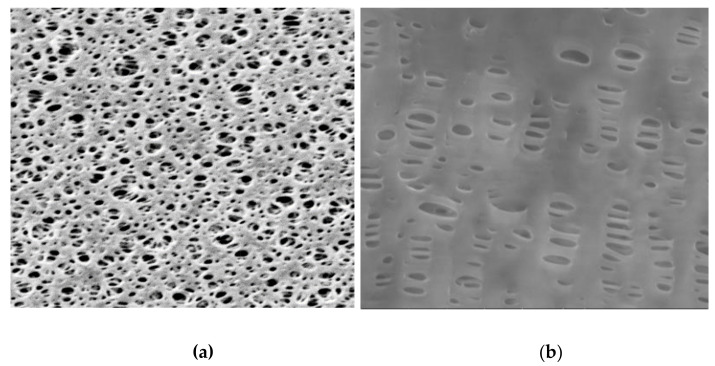
SEM photographs of virgin membrane (1000×): (**a**) PT PES membrane; (**b**) HF PP membrane.

**Figure 20:**
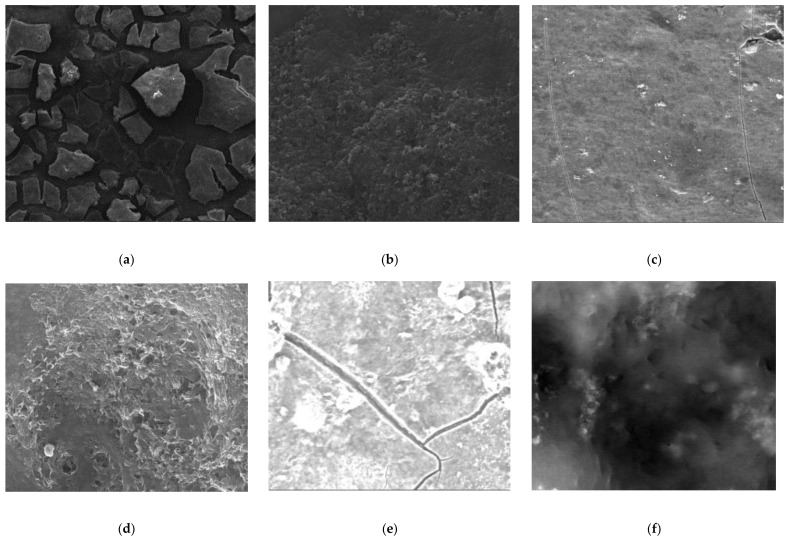
SEM images of fouled PT PES membrane: (**a**) Fe^2+^ experiments (1000×); (**b**) Fe^2+^ experiments (5000×); (**c**) Mn^2+^ experiments (1000×); (**d**) Mn^2+^ experiments (5000×); (**e**) Fe^2+^-Mn^2+^ experiments (1000×); (**f**) Fe^2+^-Mn^2+^ experiments (5000×).

**Figure 21:**
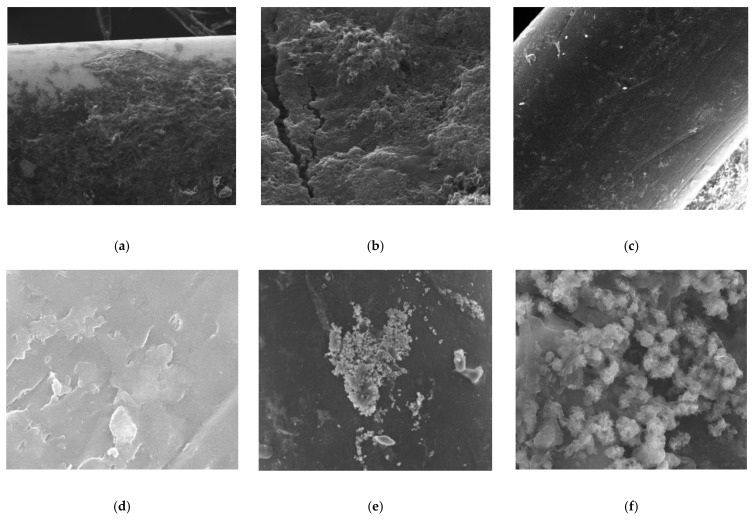
SEM images of fouled HF PP membranes (**a**) Fe^2+^ experiments (1000×); (**b**) Fe^2+^ experiments (5000×); (**c**) Mn^2+^ experiments (1000×); (**d**) Mn^2+^ experiments (5000×); (**e**) Fe^2+^-Mn^2+^ experiments (1000×); (**f**) Fe^2+^-Mn^2+^ experiments (5000×).

PT PES and HF PP membranes were investigated with a scanned electron microscope SEM/FEI-Quanta FEG 250 (Serontech, Gyeonggi-do, Korea) before and after the membrane experiments with a magnification of 1000× and 5000×. Figure 19, Figure 20, Figure 21 give SEM images of virgin and fouled PT PES and HF PP membranes.

According to Figure 20, a thick cake layer formed on PT PES membrane surface for Fe^2+^ removal experiment rather than irreversible fouling. Furthermore, a maintained porous structure indicates that iron oxide accumulated on the membrane surface rather than pores and acted as a second membrane. It can be said that this has led to low flux reduction and a positive contribution to the membrane life. On the other hand, it is disadvantageous for the mechanical behavior of the membrane [56]. The SEM images also showed that the thickness of the cake layer on the surface of the membranes was different for Fe^2+^ and Mn^2+^ removal experiments. On the other hand, foulant accumulation mainly occurred in membrane pores for the Mn^2+^ removal experiment [5].

When the fouled membrane of the Fe^2+^-Mn^2+^ experiment is examined, it can be seen that the pores are more prevalent and visible than the Mn^2+^ experiment, but denser and more open textured than the Fe^2+^ experiment. This was attributed to Fe(OH)_3_ formation retards membrane fouling via cake layer formation.

## 4. Conclusions

In this study, iron and manganese removal from water was investigated separately with aeration and aerated-submerged membrane system. Two different membranes, plate type polyethersulfone (PT PES) and hollow fiber polypropylene (HF PP) membrane, were used in the aerated-submerged membrane system.

When the results of the oxidation and aerated membrane studies were evaluated, it can be said that formed iron and manganese oxides extended Fe^2+^, especially Mn^2+^ oxidation, due to floc formation and adsorption/surface oxidation. Early oxidation studies showed that iron and manganese oxides have a catalytic effect up to 600–800 mg/L. In addition, it is observed that the catalytic effect increased up to from three to four days with the aging of oxides. However, the catalytic effect of iron oxide is greater than that of manganese oxide to remove both Fe^2+^ and Mn^2+^.

Due to the combination of all of these mechanisms with filtration proses in the aerated submerged membrane system, Fe(OH)_3_ increase Fe^2+^ and Mn^2+^ removal efficiency through surface adsorption/oxidation and the flocs it produces also grows beyond the membrane and can cause an increase in membrane productivity. It was seen that Fe(OH)_3_ caused a decrease in pressure increase, which is an indicator of membrane fouling. Besides, it can be said that iron oxide retards membrane fouling via cake layer formation on membrane surface acting as a second membrane layer.

## Figures and Tables

**Table 2:** Properties of membranes.

	HF PP	PT PES
Membrane type	Hollow Fiber-P5	Flat-MP005
Surface area, cm^2^	1.168	16
Membrane material	Polypropylene	Polyethersulfone
Pore size	0.1 μm × 0.5 μm	0.05 μm
Characteristic flux	216 L/m^2^·h	-
Pure water flux	-	400 L/m^2^·h
OD/ID	240/310 μm	-
Bursting press	>5.5 bar	-
Crash pressure	>3.5 bar	-
Temperature	-	95 °C
pH	-	0–14

**Table 3:** The effect of MnO_2_ and Fe(OH)_3_ on the oxidation of Fe^2+^ with atmospheric oxygen (Fe^2+^ = 3 mg/L, pH = 6.5, alkalinity = 2 × 10^−2^ eq/L, temperature = 25 °C).

pH	Fe^2+^ (mg/L)	MnO_2_ (mg/L)	Fe(OH)_3_ (mg/L)	k/kcat (min^–1^)	Time (min)
6.5	3	0	0	0.038	79
6.5	3	5	0	0.041	73
6.5	3	25	0	0.051	59
6.5	3	50	0	0.08	36
6.5	3	0	5	0.05	60
6.5	3	0	25	0.063	47
6.5	3	0	50	0.107	27

**Table 4:** The effect of MnO_2_ and Fe(OH)_3_ on the oxidation of Mn^2+^ with atmospheric oxygen (Mn^2+^ = 3 mg/L, pH = 9.2, alkalinity = 2 × 10^−2^ eq/L, temperature = 25 °C).

pH	Mn^2+^ (mg/L)	MnO_2_ (mg/L)	Fe(OH)_3_ (mg/L)	k/kcat (min^−1^)	Time (min.)
9.2	3	0	0	0.0169	177
9.2	3	5	0	0.0194	150
9.2	3	25	0	0.0265	113
9.2	3	50	0	0.0272	110
9.2	3	0	5	0.0195	134
9.2	3	0	25	0.0269	111
9.2	3	0	50	0.0392	76

**Table 5:** Initial and steady-state fluxes.

Membrane Type	J_W_	Fe^2+^ (90 Days)		Mn^2+^ (90 Days)		Fe^2+^-Mn^2+^ (90 Days)	
		J_d_	J_0_	J_d_	J_0_	J_d_	J_0_
PT PES	400	148.57	470.00	10.81	84.96	60.22	93.69
HF PP	216	9.48	91.79	9.37	54.70	41.53	96.85

**Table 6:** Resistance values.

Membrane Type	(Fe^2+^)				(Mn^2+^)				(Fe^2+^-Mn^2+^)			
	*R_t_* ×10^12^	*R_m_* ×10^12^ (%)	*R_p_* ×10^12^ (%)	*R_c_* ×10^12^ (%)	*R_t_* ×10^12^	*R_m_* ×10^12^ (%)	*R_p_* ×10^12^ (%)	*R_c_* ×10^12^ (%)	*R_t_* ×10^12^	*R_m_* ×10^12^ (%)	*R_p_* ×10^12^ (%)	*R_c_* ×10^12^ (%)
PT PES	1.94	0.72 37.11	0.26 13.40	0.96 49.49	26.64	0.72 2.70	7.47 28.04	18.45 69.26	4.78	0.72 15.06	1.02 21.34	3.04 63.60
HFPP	8.28	1.33 16.06	2.45 29.59	4.50 54.35	30.73	1.33 4.33	9.57 31.14	19.83 64.53	6.93	1.33 19.20	2.04 29.43	3.56 51.37

## Data Availability

The data presented in this study are available on request from the corresponding author.
